# Patient-reported Reasons for Stopping Care or Switching Clinics in Zambia: A Multisite, Regionally Representative Estimate Using a Multistage Sampling-based Approach in Zambia

**DOI:** 10.1093/cid/ciaa1501

**Published:** 2020-10-03

**Authors:** Izukanji Sikazwe, Ingrid Eshun-Wilson, Kombatende Sikombe, Laura K Beres, Paul Somwe, Aaloke Mody, Sandra Simbeza, Chama Bukankala, David V Glidden, Lloyd B Mulenga, Nancy Padian, Peter Ehrenkranz, Carolyn Bolton-Moore, Charles B Holmes, Elvin H Geng

**Affiliations:** 1 Centre for Infectious Disease Research in Zambia, Lusaka, Zambia; 2 Washington University in St Louis, St Louis, Missouri, USA; 3 London School of Hygiene and Tropical Medicine, London, United Kingdom; 4 Johns Hopkins University, Baltimore, Maryland, USA; 5 Ministry of Health, Lusaka, Zambia; 6 University of California Berkeley, Berkeley, California, USA; 7 Bill and Melinda Gates Foundation, Seattle, Washington, USA; 8 University of Alabama at Birmingham, Birmingham, Alabama, USA; 9 Georgetown University, Washington, D.C., USA

**Keywords:** retention, disengagement, reasons, HIV, Zambia

## Abstract

**Background:**

Understanding patient-reported reasons for lapses of retention in human immunodeficiency virus (HIV) treatment can drive improvements in the care cascade. A systematic assessment of outcomes among a random sample of patients lost to follow-up (LTFU) from 32 clinics in Zambia to understand the reasons for silent transfers and disengagement from care was undertaken.

**Methods:**

We traced a simple random sample of LTFU patients (>90 days from last scheduled visit) as determined from clinic-based electronic medical records from a probability sample of facilities. Among patients found in person, we solicited reasons for either stopping or switching care and predictors for re-engagement. We coded reasons into structural, psychosocial, and clinic-based barriers.

**Results:**

Among 1751 LTFU patients traced and found alive, 31% of patients starting antiretroviral therapy (ART) between 1 July 2013 and 31 July 2015 silently transferred or were disengaged (40% male; median age, 35 years; median CD4 level, 239 cells/μL); median time on ART at LTFU was 480 days (interquartile range, 110–1295). Among the 544 patients not in care, median prevalences for patient-reported structural, psychosocial, and clinic-level barriers were 27.3%, 13.9%, and 13.4%, respectively, and were highly variable across facilities. Structural reasons, including, “relocated to a new place” were mostly cited among 289 patients who silently transferred (35.5%). We found that men were less likely to re-engage in care than women (odds ratio, .39; 95% confidence interval, .22–.67; *P* = .001).

**Conclusions:**

Efforts to improve retention of patients on ART may need to be tailored at the facility level to address patient-reported barriers.

## Teaser

Structural, psychosocial, and clinical reasons for disengagement are prevalent and highly variable across facilities. Men are less likely to re-engage in care than women. Patients reporting clinical barriers have less likelihood of returning to care if they report a psychosocial barrier.

Keeping persons living with human immunodeficiency virus (HIV) on antiretroviral therapy (ART) poses a lasting and formidable challenge in the global public health response. To obtain long-term health benefits, patients must overcome barriers to physical access to care and psychosocial obstacles and navigate sometimes difficult and frustrating health systems [1]. Globally, over 20 million persons have started ART, making the magnitude of the retention challenge greater than ever [2]. Yet, HIV treatment guidelines, policies, and health services struggle to evolve quickly enough to meet the needs of the many individuals on treatment, including those with no acute need for facility-based care. Recent data from Tanzania suggest that 35% of patients are lost to follow-up (LTFU) within 2 years after starting therapy—a group that represents a mixture of those who have remained in care but whose transfers are unofficial and undocumented as well as those who genuinely stop treatment—and this figure has changed very little over the last decade [3]. The public health response to the HIV epidemic is succeeding in getting more people to start ART, but challenges remain in retaining people in care as well as in tracking those who interrupt care and encouraging them to re-engage [4].

Understanding reasons why patients stop care or change facilities can help programs understand and prioritize opportunities for improvement. While many epidemiological studies have demonstrated relationships between sociodemographic characteristics and retention, viral suppression, or mortality, these relationships generally show small effect sizes and also give us relatively little information about why certain groups have trouble staying in care [5–8]. For example, a large number of studies find that men exhibit consistently poorer retention than women in many countries [9–12]. But while sex may be a good marker for poor retention, such associations do not reveal the behavioral and psychosocial mediators of these effects—and therefore do not provide adequate resolution in our understanding to be actionable. Targeting all men for extra support would be neither efficient nor optimally effective [5, 6, 8, 13–17]. Qualitative research, on the other hand, offers a deeper understanding of barriers to engagement (eg, stigma, depression, distance) [18–20]. Yet, such studies are not designed to be quantitative nor representative and therefore are less useful for prioritizing any particular barrier identified in a population.

In this paper we present results of a study to assess a probability sample of patients LTFU from their original clinical sites in Zambia. After attempts to contact the lost patients, we collected patient-reported reasons for stopping care or switching clinics through a previously developed semi-structured survey (Supplementary Appendix 1) and coded reasons for stopping care or changing clinics. This approach allowed for numerous and nuanced barriers to engagement in HIV care to be explored with participants and offered the advantage of both a quantitative and qualitative approach. Taking a probability sample enabled epidemiological interpretation of findings and meaningful assessments of prevalence.

## METHODS

### Patients and Sampling

**Figure 1.**
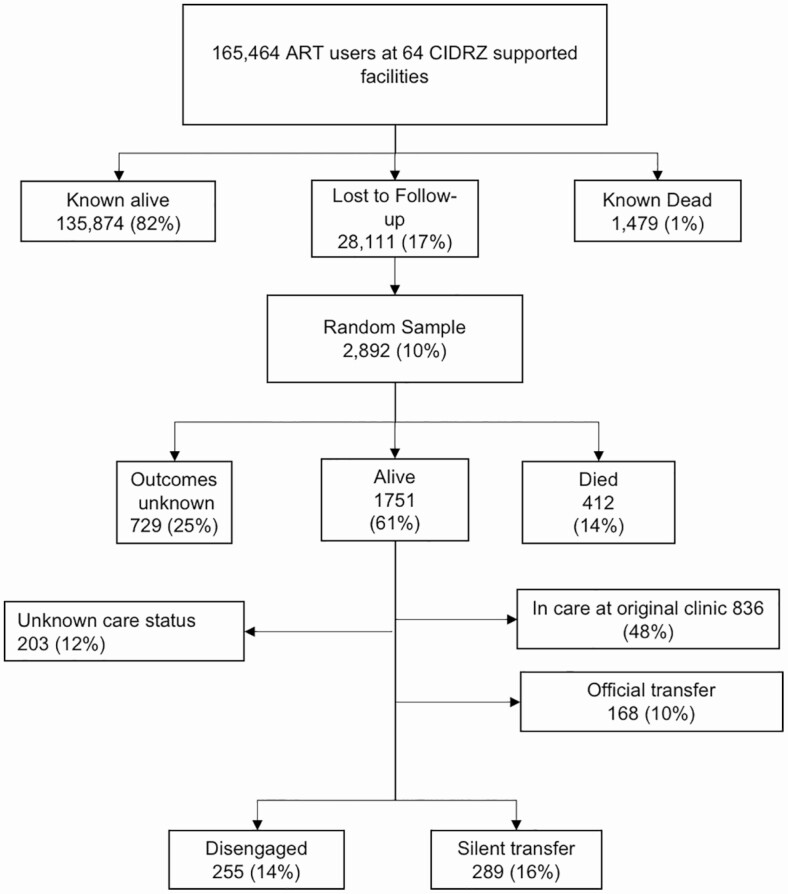
Flowchart depicting tracing outcomes among those categorized as “lost” by electronic medical record. Abbreviations: ART, antiretroviral therapy; CIDRZ, Centre for Infectious Disease Research in Zambia.

Our target population was adults with HIV who were LTFU from ART programs in Zambia. We evaluated adults with HIV 18 years or older who sought ART services during a 24-month period (August 2013 to July 2015) across 64 public health facilities in 4 provinces (Western, Lusaka, Eastern, and Southern provinces) in Zambia supported with funding from the President's Emergency Plan For AIDS Relief/Centers for Disease Control and Prevention (PEPFAR/CDC) through the Centre for Infectious Disease Research in Zambia (CIDRZ). To obtain regionally representative predictors of care status and reasons for leaving their original clinic, we used a multistage sampling approach (Figure 1), and as described previously [7, 21], we stratified sampling of sites by province and health facility level (eg, rural health center, urban health center, and hospital), resulting in 12 joint strata. The probability of selection of a health facility was proportional to its size. In the 32 selected sites, using the electronic medical record (EMR) system, we enumerated all adults 18 years or older currently on ART and who had an encounter with the facility during the previous 24-month period. Among patients LTFU, defined as at least 90 days late (in line with national guidelines) for the last visit and not known to be dead or transferred out according to the EMR, we selected a random sample of lost patients for active tracing.

### Procedures and Measurements

We obtained sociodemographic and clinical data for all patients from the EMR. We recruited peer health workers with in-depth knowledge of patient flow within facilities as well as familiarity with the surrounding communities to trace LTFU patients. The study staff initially reviewed patient paper charts and EMRs within the health facility to confirm vital and care status before proceeding to phone calls and in-person tracing within the community for those participants who truly had unknown outcomes. We defined 2 possible updated care states for patients who had stopped care at their original health facility and were identified to be alive: we considered those who remained out of care as “disengaged” and those who had re-engaged at a new facility (ie, undocumented transfer) as “silent transfers.” Tracers used a semi-structured questionnaire to collect updated care status and reasons for disengagement or silent transfer. In addition, patients who were disengaged were asked “What would have to happen for them to return to care” as an alternative way to understand the most important and tractable barriers (ie, the “sufficient cause” for return) from the patient perspectives. For all surveys, peer health workers also captured additional patient comments in free-text fields that were later categorized by study staff to correspond to original survey questions or to generate new reasons that had not been captured by the original survey. To enable policy makers and HIV program implementers to replicate our activities, we developed the Better Information for Health Toolkit (Supplementary Appendix 2).

### Analysis

We determined frequencies of patient-reported reasons for silent transfer and disengagement, categorized into domains (structural, psychosocial, and clinic-related) [22]. “Structural” reasons included challenges with access to care, such as difficulties with transport, interference due to work, or changing circumstances due to travel. “Psychosocial” reasons were related to personal difficulties, such as the need to prioritize social obligations over ART and fear of disclosure. We further classified reasons such as issues with care quality, waiting time, and disrespectful health workers as “clinic” related. We similarly characterized “what it would take to return” among the disengaged patients into structural, psychosocial, and clinic related changes. We determined frequencies of patient-reported reasons for the 3 care states and evaluated these by health facility to capture heterogeneity across sites and examined the joint distribution of reasons using Venn diagrams. We applied sampling weights to all estimates to allow the findings to represent the population from which the sample was obtained. We tested the association between reason domains and the probability of a binary outcome of silent transfer or disengagement using logistic regression. We used multiple imputation for missing demographic data (<25%), sampling weights, and predictive margins to evaluate interactions [23]. The full protocol for analysis is available in Supplementary Appendix 3.

## RESULTS

As described in previous work [7], 165 464 patients on ART received care across the 64 health facilities during the 24-month period, including 49 129 patients newly initiating ART; 1479 (1%) of patients were documented to have died; and 28 111 (17%) were LTFU. The median facility size among the 32 sampled clinics was 4734 patients (interquartile range [IQR], 3042–7502 patients). A random sample of 2892 (10%) of those LTFU were intensively tracked to ascertain their vital status (Figure 1). Of the 1751 (61%) who were found alive, 12% had an unknown care status (study team was unable to gather further information from either an informant or the patient), 48% were in care at the original health facility (83% ascertained through chart review alone and 17% through tracing), 10% had officially transferred, 16% were attending care at another facility, and 14% (n = 255) were disengaged. The characteristics of the 544 patients that had either disengaged or LTFU is described in Table 1.

**Table 1.** **Characteristics of Disengaged and Silent-transfer Patients Sampled and Surveyed**

Characteristic	Values
Age at last visit, median (IQR), years	35 (30–41)
Male gender	220 (40)
Enrollment CD4 count,^a^ median (IQR), cells/μL	239 (131–366)
Time on ART at loss to follow-up, median (IQR), days	480 (110–1295)
WHO stage at enrollment, n (%)	
Stage 1	234 (43)
Stage 2	92 (17)
Stage 3	136 (25)
Stage 4	31 (6)
Unknown	51 (9)
Province, n (%)	
Eastern	94 (17)
Lusaka	235 (43)
Southern	121 (22)
Western	94 (17)
Facility, n (%)	
Rural	100 (18)
Urban	307 (56)
Hospital	137 (25)
Disclosure, n (%)	
No	9 (2)
Yes	533 (98)
Unknown	2 (0)
Educational level, n (%)	
None	31 (6)
Lower-mid basic	199 (37)
Upper basic/secondary	240 (44)
College/university	67 (12)
Unknown	7 (1)
Marital status, n (%)	
Single	104 (19)
Married	313 (58)
Divorced	74 (14)
Widowed	49 (9)
Unknown	4 (0)
Care status, n (%)	
Silent transfer	289 (53)
Disengaged	255 (47)

N = 544.

Abbreviations: ART, antiretroviral therapy; IQR, interquartile range; WHO, World Health Organization.

^a^CD4 count missing for 103.

**Figure 2.**
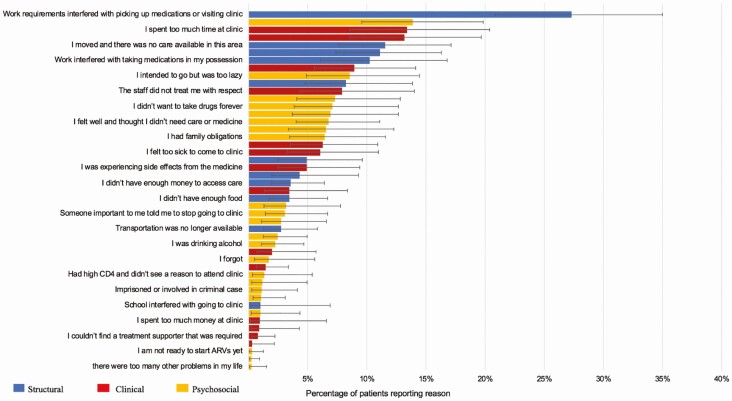
Reasons for disengagement. n = 255. Abbreviation: ARV, antiretroviral threapy.

**Figure 3.**
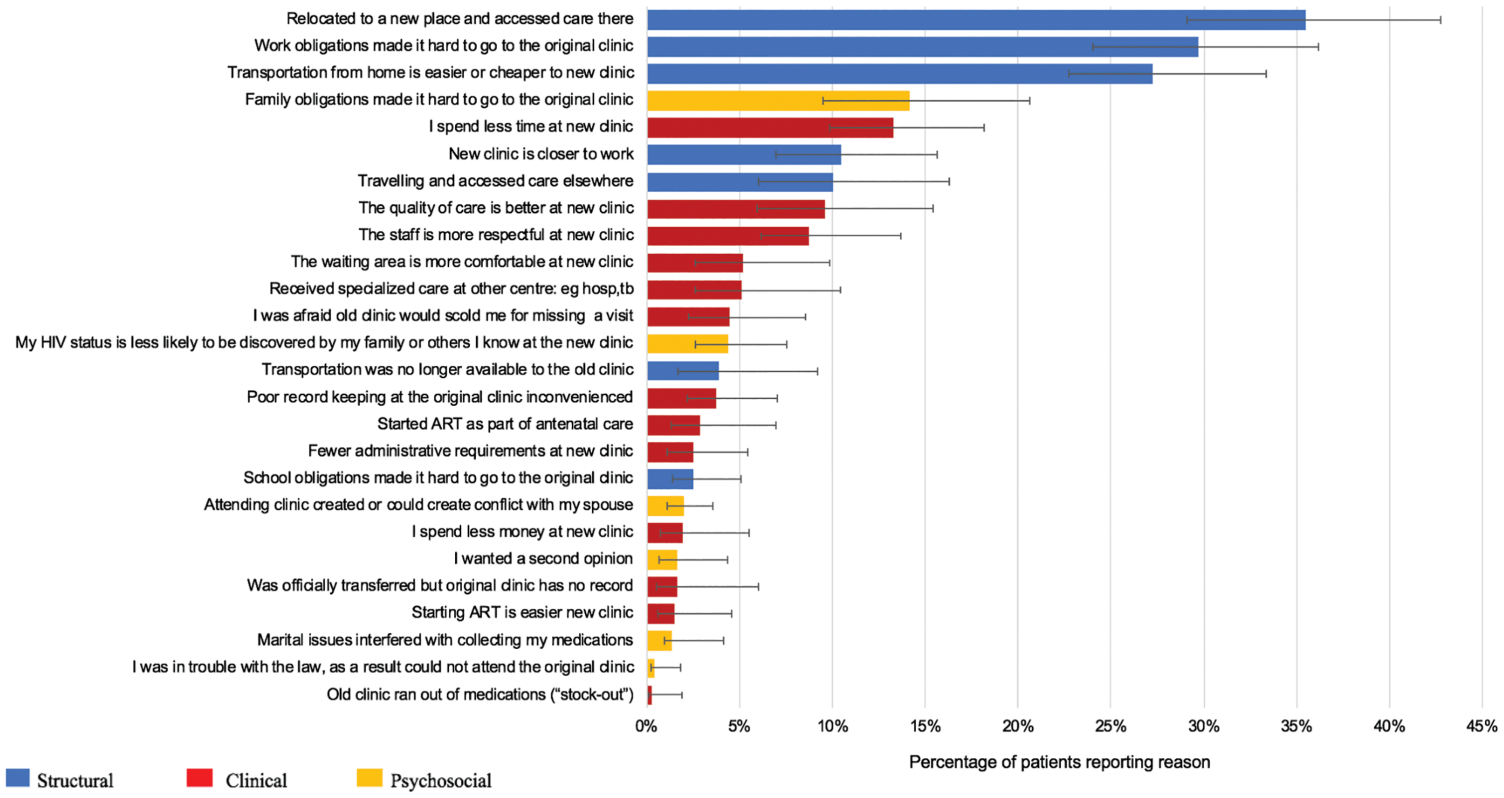
Reasons for silent transfer. n = 289. Abbreviations: ART, antiretroviral therapy; HIV, human immunodeficiency virus.

**Figure 4.**
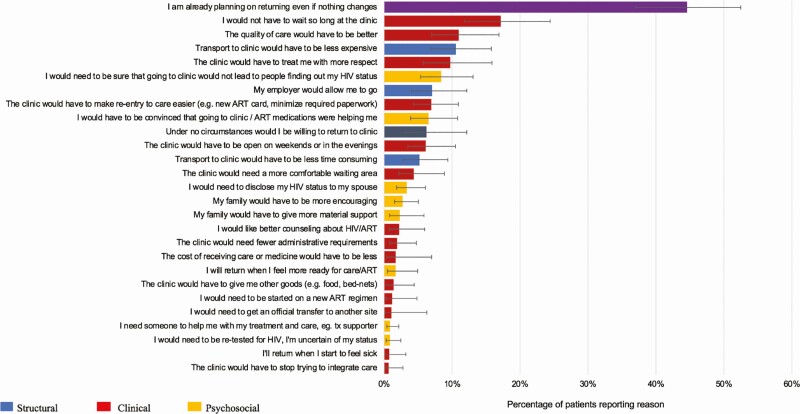
Reasons to return to care if disengaged. n = 255. Abbreviations: ART, antiretroviral therapy; HIV, human immunodeficiency virus. tx, treatment

Among 255 patients lost from their original clinics who had not re-engaged in care, structural reasons for disengagement such as “work requirements interfering with attendance” (27.3%; 95% confidence interval [CI], 20.8–35.0%) played a large role, but psychosocial reasons such as “attending clinic risked disclosure” (13.9%; 95% CI, 9.5–19.8%) were also reported frequently. Clinic-based reasons were also common among those disengaged from care (“I spent too much time at the clinic”; 13.4%; 95% CI, 8.5–20.4%) (Figure 2). The most common reasons cited among participants with undocumented transfer to a new facility were structural, including “relocated to a new place” (35.5%; 95% CI, 28.7–42.8%), “work obligations made it hard to go to the original clinic” (29.7%; 95% CI, 23.3–37.0%), and “transportation to new clinic was easier or cheaper” (27.2%; 95% CI, 21.5–33.7%). Although not as common, clinic reasons for the transfer were given (“I spend less time at the new clinic”; 13.3%; 95% CI, 8.7–4.6%) (Figure 3). When questioned on what it would take to return to care among the disengaged, clinic-based changes, such as “I would not have to wait so long at the clinic” (17.2%; 95% CI, 11.8–24.4%) and “the quality of care would have to be better” (11.0%: 95% CI, 7.0–16.9%) were highlighted. Approximately 44.6% (95% CI, 37.0–52.5%) of patients reported that they intended to return even if no changes occurred (Figure 4).

**Figure 5.**
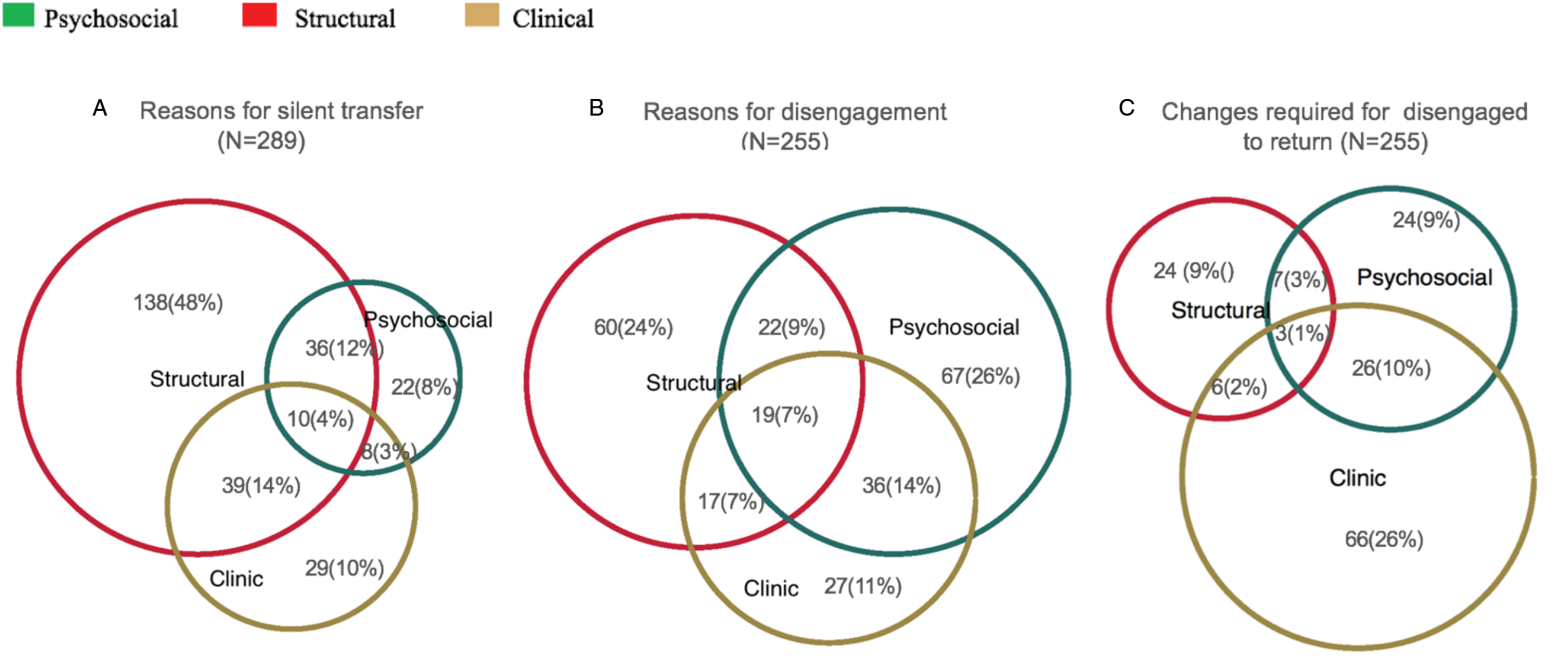
Venn diagrams depicting overlap between barrier domains.

Patients reported multiple reasons, and we represented this overlap using Venn diagrams (Figure 5). These diagrams represent the relative contribution of various reason domains and how they overlap. The median number of reported reasons for silent transfer or disengagement was 2 (IQR, 2–3), with 77% (198/255) of disengaged patients and 87% (250/289) of silent transfers reporting more than 1 reason.

**Figure 6.**
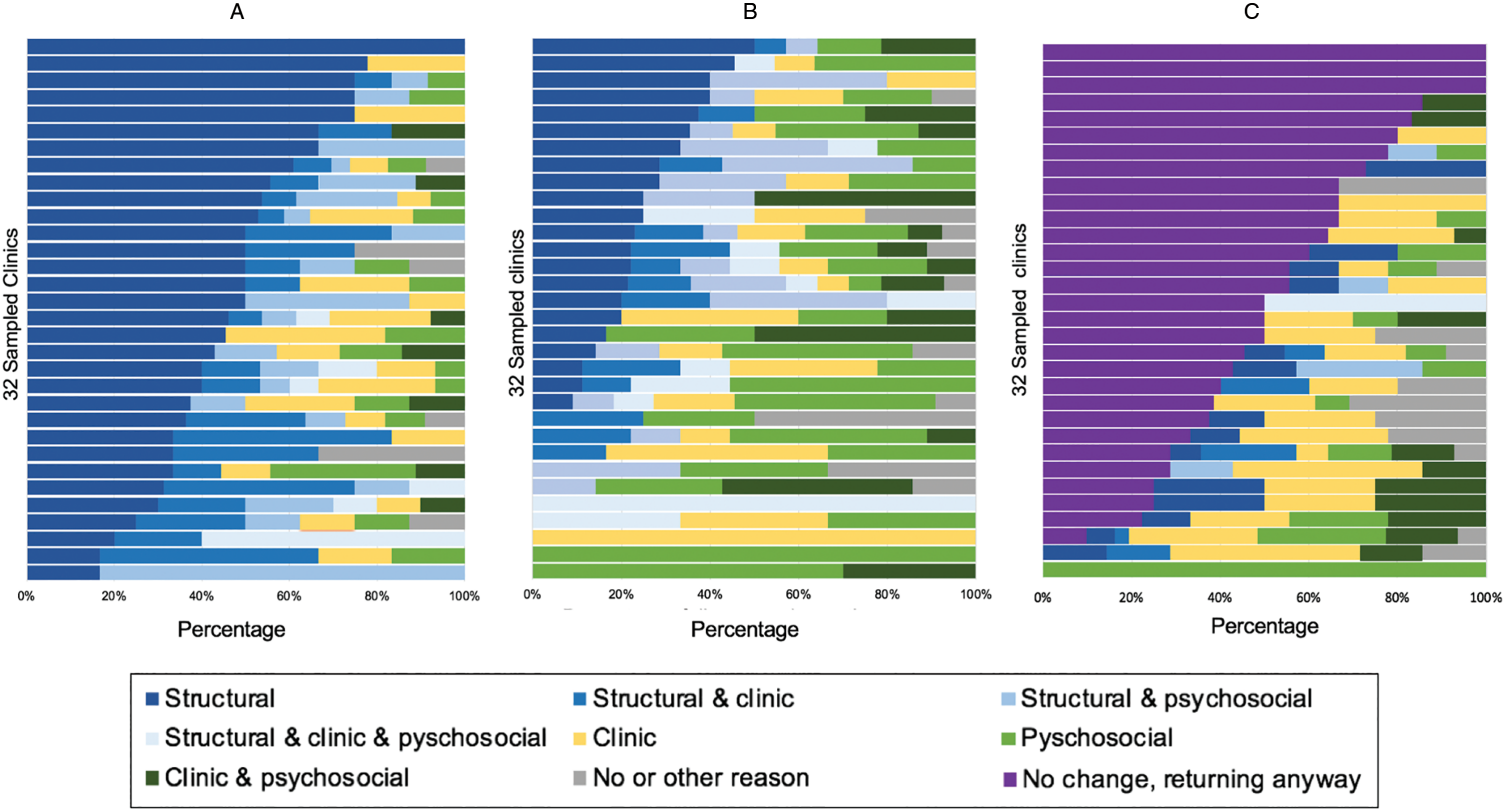
Facility-level reasons for silent transfer, n = 289 (*A*); for disengagement (participants who were found to be alive but out of care), n = 255 (*B*); and patient-reported changes required to return to care, among those disengaged, n = 255 (*C*).

A high level of heterogeneity was observed when these reasons were stratified by clinic. When we pooled facility-level data and evaluated reason domains, the predominance of structural reasons among silent transfers and psychosocial reasons among those disengaged was apparent; there was also marked heterogeneity across sites, where some sites had 100% of patients reporting a structural barrier and others reporting closer to 60% (Figure 6). This variation was seen across facilities, both for reasons for silent transfer and disengagement as well as what would be needed for return.

**Figure 7.**
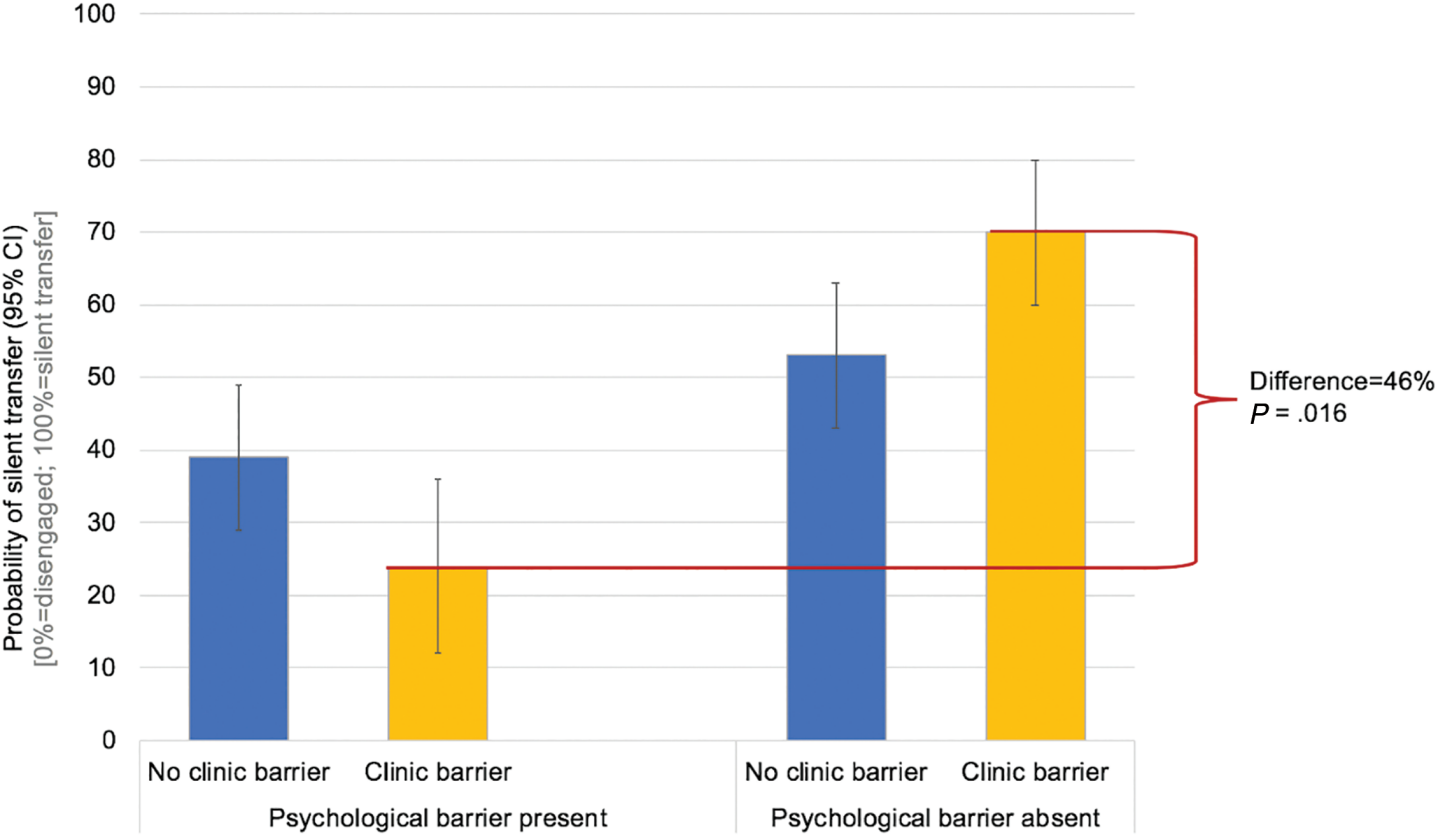
Estimated probability of re-engagement at a new facility (ie, silent transfer) among patients lost to follow-up from their original care facility (adjusted as per Table 2). Abbreviation: CI, confidence interval.

A multivariable logistic regression model was created that adjusted for other sociodemographic factors among the 544 patients lost from their original clinics during the 24-month period under study and in whom updated care status was ascertained (Table 2). After adjustment, 40.0% (95% CI, 51.4–64.6%) of men had re-engaged compared with 58.0% (95% CI, 51.4–64.6%) of women (odd ratio, .39; 95% CI, .22–.67; *P* = .001). Patients reporting a clinic-based barrier had a reduced likelihood of having returned to care if they also reported a psychosocial barrier. However, if there was a clinic-based barrier alone there was an increased probability of re-engagement. In other words, in the absence of a psychosocial barrier to care, the presence of a clinic barrier tended to drive patients away from their original clinic (but into care at another facility) compared with the remaining patients who did not have clinic reasons (73%; 95% CI, 67–79% of whom reported a structural reason) (Figure 7). However, in the patients who also had psychosocial reasons for being LTFU, a clinic barrier tended to drive people out of care all together.

**Table 2.** **Factors Associated With Being Found to Be in Care Among Those Lost to Follow-up**

Variable	Odds Ratio	Lower CI	Upper CI	*P*
Reported reasons for care status				
Clinic reason				
No psychosocial reason	2.12	.50	3.73	.008^a^
Psychosocial reason	0.45	.06	.84	
Psychosocial reason				
No clinic reason	.55	.18	.92	
Clinic reason	.12	.01	.22	
Structural reason	2.86	1.52	5.41	.001
Patient and facility characteristics				
Male gender	.39	.22	.67	.001
Age (per 10 years)	1.23	.83	1.53	.443
CD4 count (per 100 mmol)	.91	.80	1.04	.154
WHO stage				
1	1.00	…	…	.969
2	.97	.46	2.03	
3	.89	.43	1.81	
4	1.04	.35	3.14	
Time on ART (per year)	1.02	.89	1.16	.769
Province				
Lusaka	1.00	…	…	.017
Eastern	1.78	.87	3.65	
Southern	3.11	1.53	6.34	
Western	2.13	.93	4.88	
Facility size (per 100 patients)	1.06	.80	3.59	.165
Facility type				
Urban	1.00	…	…	
Rural	1.70	.80	3.59	.372
Hospital	1.14	.61	2.10	
Education status				
None	1.00	…	…	.085
Lower to mid-basic	1.10	.20	6.12	
Upper-basic to secondary	2.04	.36	11.45	
College/university	2.84	.45	17.86	
Ever married				
Married	1	…	…	.717
Single	.87	.44	1.73	
Divorced	.66	.33	1.35	
Widowed	1.26	.47	3.38	
Ever disclosed HIV status				
Yes	1	…	…	.490
No	.54	.09	3.10	

N = 544. Logistic regression model with inverse probability sampling weights applied and multiple imputation for missing predictor variables.

Abbreviations: ART, antiretroviral therapy; CI, 95% confidence interval; WHO, World Health Organization.

^a^*P* value for interaction between clinic and psychosocial reasons.

## DISCUSSION

In a representative sample of patients who were LTFU from their original facility site, we found about half of the 544 patients included in the analysis were in care at a new facility and the rest were out of care. This result indicates both a need for safe, efficient, facilitated transfer when warranted across facilities, as well as efforts to identify and re-engage people who have stopped care entirely. Structural barriers to care were most prevalent among people who had been LTFU who subsequently re-engaged at a new facility, whereas psychosocial reasons were more common among people who had been LTFU and remained out of care. The presence of both clinical and psychosocial barriers to care was associated with a higher probability of being out of care than either alone. Across facilities, the prevalences of reasons for both silent transfer and stopping care were markedly varied, indicating that efforts to improve retention of patients on ART may need to be tailored at the facility level to address particular context and the barriers faced by its patient population. Finally, despite these barriers, the vast majority of patients who had been LTFU indicated a desire to return to care at their original clinic even if nothing changed. This finding may represent social desirability bias, which has been shown to influence self-report of both HIV treatment and risk behaviors [24–26] or the gap between patient intention and health behavior [27, 28]. It could be a feature of the in-person interviewing approach utilized in the study, which also allowed for encouragement to return to care [29]. Prior work and qualitative evidence from this study demonstrate that complex factors interact to influence patient HIV engagement [19]. Individual, social, and structural barriers may interrupt the pathway from intention to action, while resources such as self-efficacy, planning, and locus of control may mediate behavioral outcomes, particularly in a chronic disease setting such as HIV [30]. The barriers faced by patients may speak to the insights from behavioral economics of the ways that people discount activities that have near-term costs but are recognized to have long-term benefits [31]. Patients may, for example, repeatedly defer ART pick-up and maintain drug adherence and ultimately long-term HIV viral load suppression to pursue engagements with immediate rewards despite appreciating the benefits of ART.

This paper suggests that the reasons for leaving a facility determine whether a patient enters care elsewhere or stops care altogether. Structural barriers to access, such as costs, time, and transportation, tend to drive people away from a particular facility, but not out of care altogether. The fact that many people reporting these reasons re-entered care elsewhere suggests that they continued to want to receive care and resumed ART when they could overcome those barriers. Although this may reflect social desirability bias [22], these data could also support the finding that, overall, patients who are “lost to follow-up” often do return to services after a period of lapse, which is reflected in data from cohorts in South Africa and elsewhere [32–35] and suggests that patients who disengage often intend to return once circumstances change [6, 20].

In contrast, patients who reported psychosocial reasons for stopping care at the original facility, such as stigma or giving up, were unlikely to resume care elsewhere. This suggests that psychosocial factors are more fixed, and thus drive people not so much away from a given facility as away from care altogether. As such, patients who have these barriers can be seen as a population with more intractable problems and therefore who need a very different type of re-engagement strategy. Whereas those who simply can’t get there could be re-engaged with logistics, these patients need psychosocial counseling and support. One nuanced finding was an interaction between the presence of clinic-based and psychosocial reasons for disengaging: those patients with psychosocial reasons, and who are less likely to engage in care already, are further less likely to re-engage, suggesting the presence of a vulnerable population requiring identification and facilitated care. This interaction between psychosocial and clinic barriers may further explain the predominance of the clinic-based recommendations cited when disengaged patients were asked what changes would need to occur for them to return to care.

Patient-reported reasons are critical to understanding both general and local reasons for failures of retention. These patients-reported reasons are far more predictive than sociodemographic factors such as age and sex. For example, even though men are less likely to re-engage in care, those with only a clinic-based issue are 70% likely to re-engage, whereas those with a clinic-based and a psychosocial reason are only 20% likely to re-engage—a 50% absolute risk difference. This spread indicated indirectly that these questions and categories are highly valid (since misclassification usually biases toward the null). Given the heterogeneity across the clinic, the true lesson here is that HIV programs should undertake these queries among those who are lost and traced.

This study is not without limitations. From a sampling point of view, we believe these reasons are widely representative, but we had incomplete response rates as only 61% of those sought were contacted and may not be fully representative of the target population. We found fewer patients in urban, compared with rural, areas and fewer from Lusaka Province, the national capital, compared with other provinces. Thus, our reasons may be less representative of mobility and other reasons associated with the urban experience. The patient-reported reasons provide a quantifiable and nuanced picture of the barriers to care, but could be influenced by social desirability bias, driven in part by the presence of the tracers. We did not undertake inverse probability weights to counteract nonresponse as we felt that the additional analytical layers could obscure the transparency of the message.

Our study shows that the majority of disengaged patients report multiple reasons for disengagement. Consistent with the qualitative work in this same cohort [18, 19], the results demonstrate the interactive nature of factors influencing care engagement and support the need for patient-centered services that address the patient as a “whole person” in their biological, social, and structural contexts. HIV program implementers and policy makers may need to consider the array of patients’ reasons for disengagement as they design and scale-up patient-retention strategies with attention to the community’s needs and the clinic’s capacity to improve long-term patient engagement in services.

## Supplementary Data

Supplementary materials are available at Clinical Infectious Diseases online. Consisting of data provided by the authors to benefit the reader, the posted materials are not copyedited and are the sole responsibility of the authors, so questions or comments should be addressed to the corresponding author.

ciaa1501_suppl_Supplementary_Figure_S1

ciaa1501_suppl_Supplementary_Figure_S2

ciaa1501_suppl_Supplementary_Figure_S3

ciaa1501_suppl_Supplementary_Figure_S4

ciaa1501_suppl_Supplementary_Appendix_S1

ciaa1501_suppl_Supplementary_Appendix_S2

ciaa1501_suppl_Supplementary_Appendix_S3

## Notes

***Acknowledgments.*** The authors thank the participants and staff from the facilities in Zambia who took part in this study. Without them and our nonmedical interviewers, this study would not have been possible.

***Financial support.*** This work was supported by the Bill & Melinda Gates Foundation (grant number OPP1105071 to I. S., E. H. G., and C. B. H.); the University of California, San Francisco–Gladstone Institute of Virology & Immunology Center for AIDS Research, Implementation Science Working Group, through the National Institutes of Health (NIH) (grant number P30 AI027763); NIH grant K24 AI134413 (to E. H. G.); and the Johns Hopkins University Center for AIDS Research (National Institute of Allergy and Infectious Diseases (grant number P30 AI094189 to C. B. H.).

***Potential conflicts of interest.*** A. M. and D. V. G. report grants from the NIH during the conduct of the study. L. K. B. reports consultancy fees from the Bill & Melinda Gates Foundation during the conduct of the study. C. B. H. reports grants from the Bill & Melinda Gates Foundation during the conduct of the study and personal fees from the Bill & Melinda Gates Foundation outside the submitted work. P. E. is an employee of the Bill & Melinda Gates Foundation. All other authors report no potential conflicts. All authors have submitted the ICMJE Form for Disclosure of Potential Conflicts of Interest. Conflicts that the editors consider relevant to the content of the manuscript have been disclosed.
