# Supplementary material for: Patient-reported Reasons for Stopping Care or Switching Clinics in Zambia: A Multisite, Regionally Representative Estimate Using a Multistage Sampling-based Approach in Zambia
Source: Clin Infect Dis. 2020 Oct 3;73(7):e2294–302. doi: 10.1093/cid/ciaa1501 (PMC8492131; doi:10.1093/cid/ciaa1501)
Supplement: ciaa1501_suppl_Supplementary_Figure_S1 [file ciaa1501_suppl_supplementary_figure_s1.pdf]

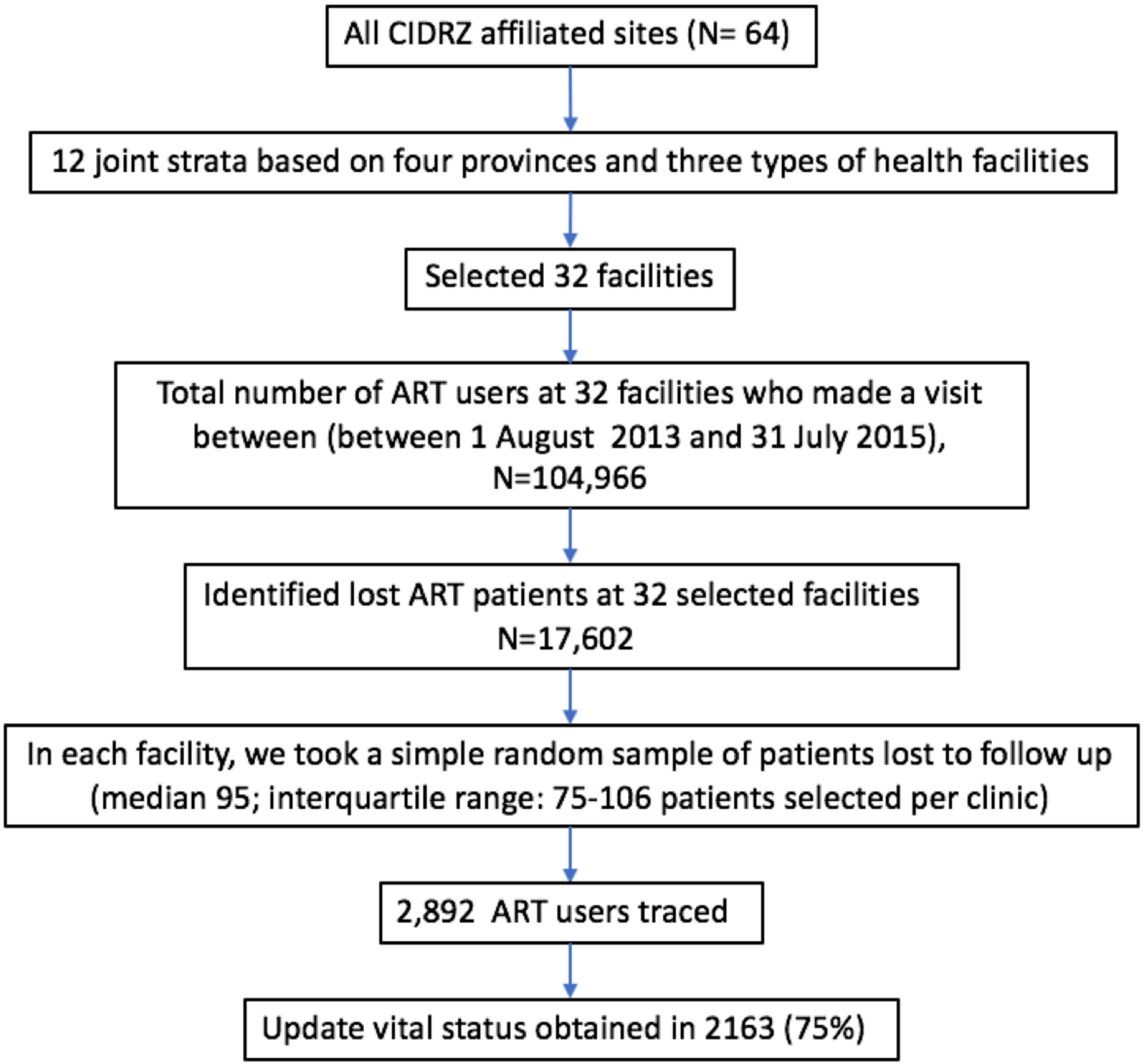
All CIDRZ affiliated sites (N= 64)
12 joint strata based on four provinces and three types of health facilities
Selected 32 facilities
Total number of ART users at 32 facilities who made a visit between (between 1 August 2013 and 31 July 2015), N=104,966
Identified lost ART patients at 32 selected facilities N=17,602
In each facility, we took a simple random sample of patients lost to follow up (median 95; interquartile range: 75-106 patients selected per clinic)
2,892 ART users traced
Update vital status obtained in 2163 (75%)
