# Supplementary figures and images for: Patient-reported Reasons for Stopping Care or Switching Clinics in Zambia: A Multisite, Regionally Representative Estimate Using a Multistage Sampling-based Approach in Zambia
Source: Clin Infect Dis. 2020 Oct 3;73(7):e2294–302. doi: 10.1093/cid/ciaa1501 (PMC8492131; doi:10.1093/cid/ciaa1501)

# Reasons for disengagement (N=255)

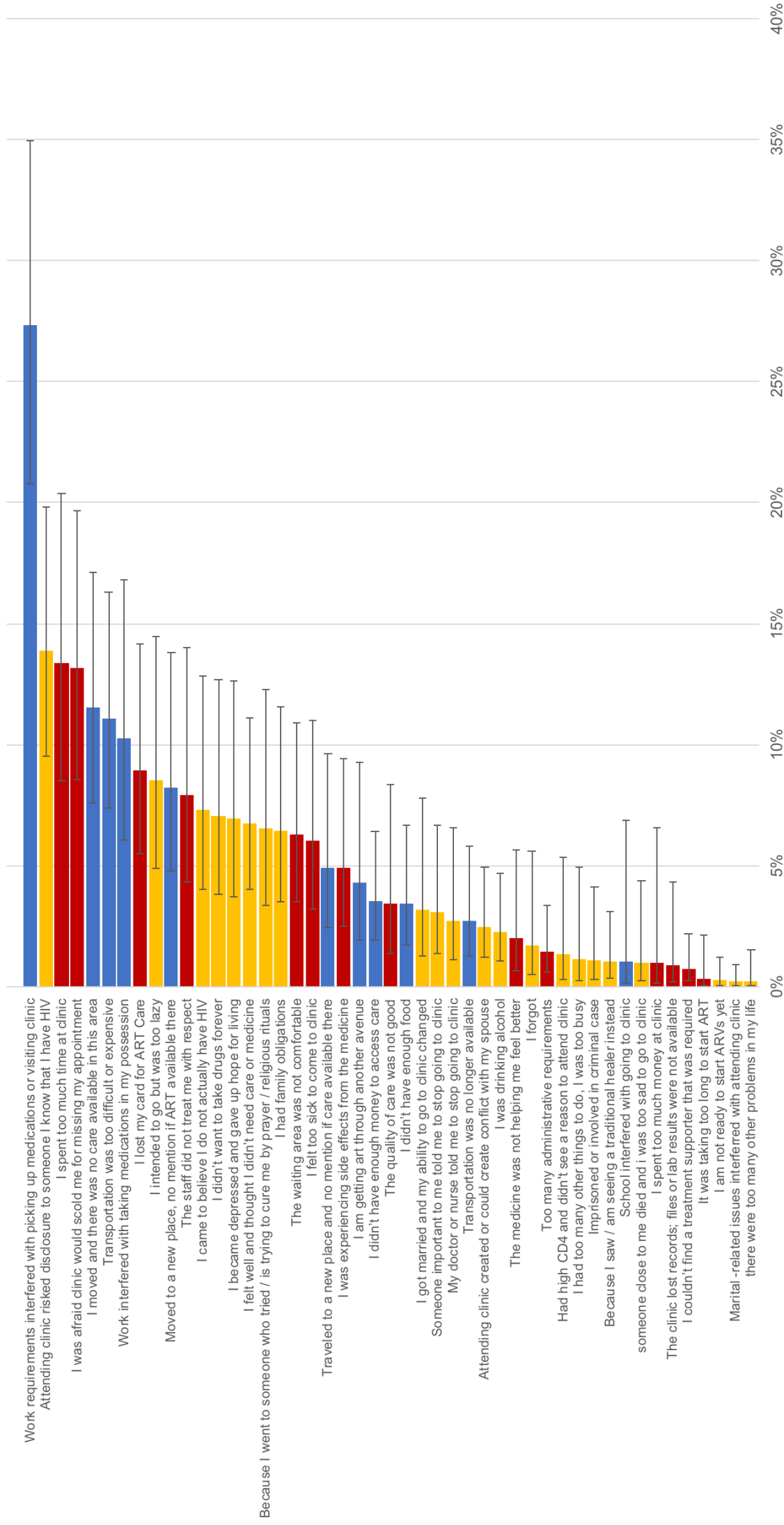

Percentage of patients reporting reason

Supplement: ciaa1501_suppl_Supplementary_Figure_S2 [file ciaa1501_suppl_supplementary_figure_s2.pdf]

## Reasons for silent transfer (N=289)

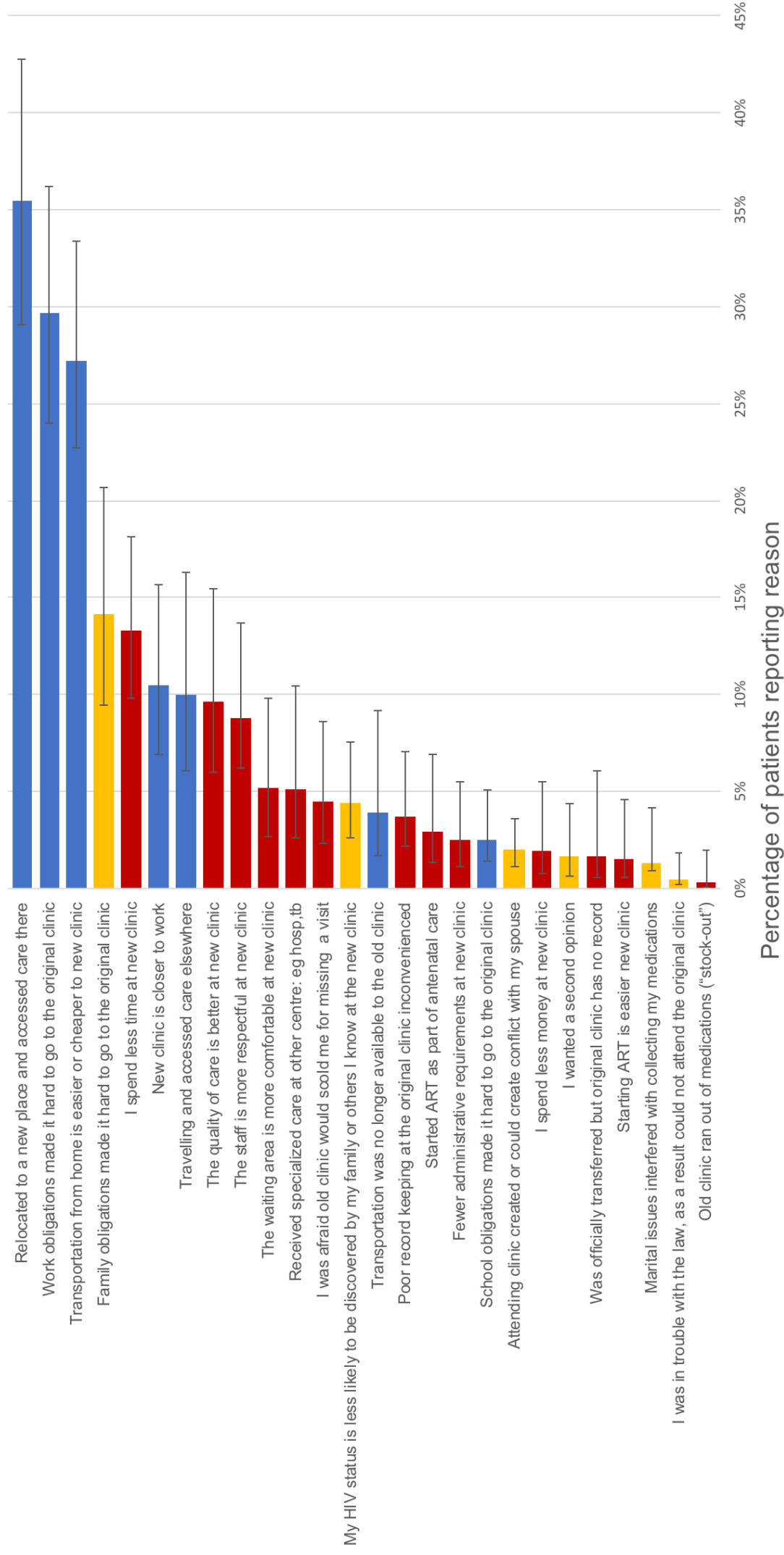

Supplement: ciaa1501_suppl_Supplementary_Figure_S3 [file ciaa1501_suppl_supplementary_figure_s3.pdf]

## Reasons to return if disengaged (N=255)

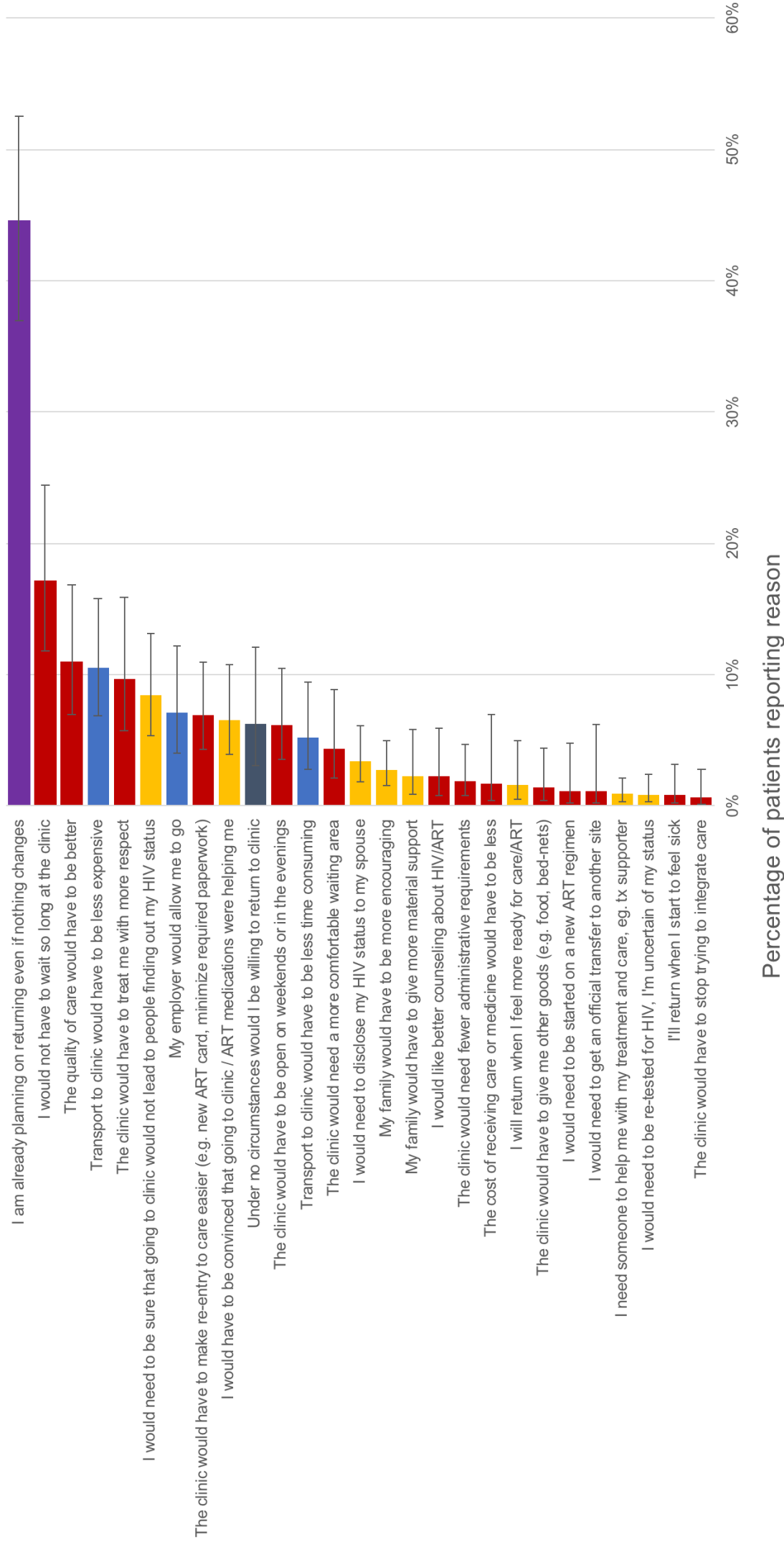

Supplement: ciaa1501_suppl_Supplementary_Figure_S4 [file ciaa1501_suppl_supplementary_figure_s4.pdf]
