# Supplementary material for: Patient-reported Reasons for Stopping Care or Switching Clinics in Zambia: A Multisite, Regionally Representative Estimate Using a Multistage Sampling-based Approach in Zambia
Source: Clin Infect Dis. 2020 Oct 3;73(7):e2294–302. doi: 10.1093/cid/ciaa1501 (PMC8492131; doi:10.1093/cid/ciaa1501)
Supplement: ciaa1501_suppl_Supplementary_Appendix_S1 [file ciaa1501_suppl_supplementary_appendix_s1.pdf]

LOST TO FOLLOW-UP TRACING FORM
MODULE B: TRACING OUTCOMES (v.2015.5.12)

B.0.1. Staff ID Number:		B.0.2. Name of patient	
B.0.3. Province:		B.0.4. Clinic name:	
B.0.5. Patient ART number:		B.0.6. Last visit date recorded in patient record:	☐☐ - ☐☐☐ - ☐☐
B.0.7. Last ART status as documented in clinic records	○ Never started three drug antiretroviral therapy (excludes limited-duration regimens for PMTCT alone)<br>○ Has ever started ART (including those who have subsequently stopped)		
B.0.8. Tracing outcome	○ Communicated with patient → go to **SECTION B1**<br>○ Communicated with informant(s) who knows the patient and not directly with patient → go to **SECTION B.2**<br>○ Attempted but unable to communicate with patient or informant → go to **SECTION B.3**		

SECTION B1: TRACER COMMUNICATES WITH PATIENT

B1.1 As of this interview, has field tracing ever occurred for this patient in this study?		○Yes<br>○No	
B1.2. Interview type. *[Pick one]*	☐ in-person ☐ phone	B1.3. Patient interview date.	☐☐ - ☐☐☐ - ☐☐<br>DAY(*dd*) MONTH (*mmm*) YEAR (*yy*)

B1.4. Confirm identity of patient using as many identifiers as possible. *Mark all that apply:*

☐ Name ☐ Age ☐ Sex ☐ Height ☐ Location or residence ☐ Occupation ☐ Clinic number or medical papers ☐ Marital Status ☐ Common Name

B1.5. Have you seen any doctor, nurse or other professional health worker (e.g., pharmacist) for the monitoring or treatment of HIV since your last visit we have on file which was [X date] at the [original clinic]?

○ Yes, I have been back to the original clinic since that date and I have not visited a new clinic in that time period	B1.5.A. What was date of last visit at original clinic as reported by patient?	☐ patient refused → skip to B1.6<br>☐ completely unknown → skip to B1.6<br>☐ exactly date known → specify<br>☐ approximate date known → specify	☐☐ - ☐☐☐ - ☐☐<br>DAY (dd) MONTH (mmm) YEAR (yy)	
○ Yes, I have visited a new care site (e.g., facility, mobile or community based treatment setting) since the last visit at the original clinic	B1.5.B. What was the date of the most recent encounter at that new care site?	☐ patient refused → skip to B1.5.C<br>☐ completely unknown → skip to B1.5.C<br>☐ exactly date known → specify<br>☐ approximate date known → specify	☐☐ - ☐☐☐ - ☐☐<br>DAY (dd) MONTH (mmm) YEAR (yy)	
	B1.5.C. What was the date of first encounter at a new care site after the last visit at [original] clinic?	☐ patient refused → skip to B1.5.D<br>☐ completely unknown → skip to B1.5.D<br>☐ exactly date known → specify<br>☐ approximate date known → specify	☐☐ - ☐☐☐ - ☐☐<br>DAY (dd) MONTH (mmm) YEAR (yy)	
	B1.5.D. What is the name of new provider organization / program	☐ patient refused → skip to B1.5.E<br>☐ completely unknown → skip to B1.5.E<br>☐ organization name known → specify		
	B1.5.E. What is the patient's ART number at the new provider?	☐ patient refused → skip to B1.5.F<br>☐ completely unknown → skip to B1.5.F<br>☐ ART number known → specify	Specify new ART number:___________________	
	B1.5.F. Did you run out of medication between your last visit at the [original clinic] and first visit at any new care site? (only applies to those on ART at last visit)		○ Yes<br>○ No →skip to B1.5.F<br>○ Refused →skip to B1.5.F	
	B1.5.F.1. How many days approximately?	☐ patient refused → skip to B1.5.G	☐☐☐ days	
	B1.5.G Why did you switch your care from [original clinic] to [new clinic]? *[mark all that apply]*			

Structural

☐ Transportation from home is easier or cheaper to new clinic.
☐ Transportation was no longer available to the old clinic
☐ New clinic is closer to work.
☐ Work obligations made it hard to go to the original clinic
☐ School obligations made it hard to go to the original clinic

Psychosocial

Clinic Factor

☐ Old clinic ran out of medications ("stock-out").
☐ The staff is more respectful at new clinic.
☐ The quality of care is better at new clinic.
☐ The waiting area is more comfortable at new clinic.
☐ I was afraid old clinic would scold me for missing a visit
☐ I received more goods (i.e., food, bednets) at new clinic
☐ I spend less money at new clinic.
☐ I spend less time at new clinic.

☐ Family obligations made it hard to go to the original clinic
☐ Attending clinic created or could create conflict with my spouse.
☐ My HIV status is less likely to be discovered by my family or others I know at the new clinic.

☐ Declines to answer
☐ Other, specify

☐ Starting ART is easier new clinic.
☐ Treatment supporter not required at new clinic.
☐ Fewer administrative requirements at new clinic.
☐ Poor record keeping at the original clinic inconvenienced me

Describe details of patient's response:	

○ No, I have not visited any new site nor seen any health worker (e.g., doctor nurse, or pharmacist) for HIV care.

B1.5.H. Why did you stop going to **any** clinic for your HIV care? [mark all that apply]

Structural
☐Transportation was too difficult or expensive.
☐Transportation was no longer available
☐ I moved and there was no care available in this area
☐ I didn't have enough money to access care.
☐ Work requirements interfered with picking up medications or visiting clinic
☐ Work interfered with taking medications in my possession.
☐ I didn't have enough food.

Psychosocial
☐ I had family obligations.
☐ I came to believe I do not actually have HIV
☐ I became depressed and gave up hope for living
☐ Attending clinic created or could create conflict with my spouse.
☐ Attending clinic risked disclosure to someone I know that I have HIV.
☐ Someone important to me told me to stop going to clinic.
☐ My doctor or nurse told me to stop going to clinic.
☐ Because I went to someone who tried / is trying to cure me by prayer / religious rituals.
☐ Because I saw / am seeing a traditional healer instead
☐ I got married and my ability to go to clinic changed
☐ I was drinking alcohol
☐ I intended to go but was too lazy
☐ I forgot

Clinic Factor
☐ The clinic ran out of medications ("stock-out").
☐ The staff did not treat me with respect
☐ The quality of care was not good.
☐ The waiting area was not comfortable.
☐ I was afraid clinic would scold me for missing my appointment.
☐ I couldn't find a treatment supporter that was required.
☐ Too many administrative requirements.
☐ I stopped receiving goods (i.e food, bednets) at clinic.
☐ I spent too much time at clinic.
☐ I spent too much money at clinic
☐ It was taking too long to start ART.
☐ I lost my card for ART Care

Medical
☐ I felt too sick to come to clinic.
☐ The medicine was not helping me feel better.
☐ I was experiencing side effects from the medicine.
☐ I felt well and thought I didn't need care or medicine.
☐ I didn't want to take drugs forever.
☐ I was taking too many pills a day.

☐ Declines to answer
☐ Other, specify:

Describe details of patient's response:	

B1.5.I. What would have to happen for you come back to care at **any** clinic? [mark all that apply]

Structural
☐ Transport to clinic would have to be less expensive
☐ Transport to clinic would have to be less time consuming
☐ My employer would allow me to go
☐ My school schedule would have to be more flexible
☐ My family would have to give more material support

Psychosocial
☐ My family would have to be more encouraging
☐ I would have to be convinced that going to clinic / ART medications were helping me
☐ I would need to be sure that going to clinic would not lead to people finding out my HIV status
☐ I would need to disclose my HIV status to my spouse

☐ Declines to answer
☐ Other, specify:

Clinic based
☐ The clinic would have to treat me with more respect
☐ I would not have to wait so long at the clinic
☐ The quality of care would have to be better
☐ The clinic would have to be open on weekends or in the evenings
☐ The cost of receiving care or medicine would have to be less
☐ The clinic would have to give me other goods (e.g. food, bed-nets)
☐ The clinic would need a more comfortable waiting area
☐ The clinic would need fewer administrative requirements
☐ The clinic would have to make re-entry to care easier (e.g. new ART card, minimize required paperwork)

Other
☐ Under no circumstances would I be willing to return to clinic
☐ I am already planning on returning even if nothing changes

Describe details of patient's response:	

○ Patient refused or cannot answer

B1.6 Approximately how long would it take you to get to the last clinic you attended (your current clinic if in-care) from your usual residence?	☐☐:☐☐<br>Hours *(hh)* : Minutes *(mm)*

If this is a phone only interview (phone ticked for B1.2), stop here and skip to Section B.4.

B1.7. ***This question is only for <u>living</u> patients not yet started on ART at original clinic. Others GO TO B1.8***
Did you ever start taking antiretroviral medications?

○Yes	B1.7.A. When did you start?	☐☐ - ☐☐☐ - ☐☐<br>DAY(*dd*) MONTH (*mmm*) YEAR (*yy*)	○ No → **Go to B1.9**

B1.8. Have you taken antiretroviral medicine (defined as any ART) in the last 14 days? (Applies only if ART status is "ever started ART" or if answer to B1.7.A is "Yes")

○Yes

B1.8.A. Where did you get the medicine? *Mark all that apply*
- ☐ Original clinic
- ☐ Private physician
- ☐ New Clinic
- ☐ Friend or family member
- ☐ Other, specify:

B1.8.B. Can you show me or name your medicines?
- ○ Yes, → *Record the medicines*
- ○ No, patient unable to name or show medicines

1. 2. 3. 4.

○No

B1.8.C. When did you last take ART?
☐☐ - ☐☐☐ - ☐☐
DAY(*dd*) MONTH (*mmm*) YEAR (*yy*)

B1.8.D. Why did you stop ART? *Answer both coded and descriptive responses*

Coded responses: *Mark all that apply*

<u>Access to Care</u>
- ☐ Transportation was too difficult or expensive.
- ☐ I didn't have enough money to access care.
- ☐ I spent too much time at clinic.
- ☐ Too many administrative requirements.
- ☐ I moved

<u>Work and Family</u>
- ☐ Work interfered with picking up medications or visiting clinic for review.
- ☐ Work interfered with taking medications.
- ☐ I had family obligations.
- ☐ Family conflict prevented attending clinic.
- ☐ Attending clinic risked disclosure to my family that I had HIV.

<u>Medical</u>
- ☐ I felt too sick to take the medicines.
- ☐ The medicine was not helping me feel better.
- ☐ I was experiencing side effects from the medicine.
- ☐ I felt too sick to come to clinic.
- ☐ I felt well and thought I didn't need care.
- ☐ I didn't want to take drugs forever.
- ☐ I was taking too many pills a day.
- ☐ I didn't have enough food.
- ☐ I was drinking alcohol.

<u>Clinic Factor</u>
- ☐ The clinic ran out of medications ("stock-out").
- ☐ The staff was not respectful.
- ☐ The care was not good.
- ☐ The waiting area was not comfortable.
- ☐ Attending clinic risked disclosure that I had HIV.
- ☐ I stopped receiving goods (i.e food, bednets) at clinic.

<u>Alternative Treatment and Advice</u>
- ☐ A family member or other important person told me to stop taking ART.
- ☐ My doctor or nurse told me to stop taking ART.
- ☐ Because I went to someone who tried / is trying to cure me by prayer / religious rituals.
- ☐ Because I saw / am seeing a traditional healer instead.
- ☐ I no longer believe I have HIV/ I believe I've been cured

- ☐ Declines to answer
- ☐ Other, specify:

Describe details of patient's response:

B1.9. Is the usual residence in the catchment area of this clinic?

○ Yes
○ No

B1.9A Did you ever spend more than 1 month consecutively away from their usual residence in the last year

○ Yes
○ No
○ Refused

B1.10. Have you disclosed your HIV status to anyone?

○ Yes
○ No →skip to B1.11
○ Declines to answer→skip to B1.11

B1.10.A. Who have you disclosed your HIV status to? [mark all that apply]

☐ Spouse/Partner
☐ Other family member
☐ Friend
☐ Co-worker
☐ Employer
☐ Other: ________________________
☐ Declines to answer

B1.11. Has anyone from the clinic contacted you either by phone or in person to encourage you to return to clinic after you had missed an appointment?

☐ N/A – the patient denies ever having missed a visit→go to **B1.14**
☐ Yes
☐ No →go to **B1.14**
☐ Declines to answer→go to **B1.14**

B1.11.A. About how many times in total has someone from the clinic contacted you either by phone or in person to encourage you to return to clinic after missing an appointment?

☐☐ times

B1.12.In the past 6 months, have you seen a traditional healer?

○ Yes
○ No
○ Declines to answer

B1.13. In the past 6 months, have you used any herbal remedies for HIV?

○ Yes
○ No →skip to B1.16
○ Declines to answer→skip to B1.16

B1.13.A Which herbal remedies have you used? If used, also tick how often it was used.

☐ Sondashi Formula	☐ Once	☐ 1x/month	☐1x/week	☐ Daily	☐ Unknown	☐ Other (specify):
☐ African Potato	☐ Once	☐ 1x/month	☐1x/week	☐ Daily	☐ Unknown	☐ Other (specify):
☐ Selenium supplement	☐ Once	☐ 1x/month	☐1x/week	☐ Daily	☐ Unknown	☐ Other (specify):
☐ Tebusha	☐ Once	☐ 1x/month	☐1x/week	☐ Daily	☐ Unknown	☐ Other (specify):
☐ Back of Mubuyu tree	☐ Once	☐ 1x/month	☐1x/week	☐ Daily	☐ Unknown	☐ Other (specify):
☐ Crocodile Fats	☐ Once	☐ 1x/month	☐1x/week	☐ Daily	☐ Unknown	☐ Other (specify):
☐ Moringa/Green Powder/Oleifera)	☐ Once	☐ 1x/month	☐1x/week	☐ Daily	☐ Unknown	☐ Other (specify):
☐ Other (specify):____	☐ Once	☐ 1x/month	☐1x/week	☐ Daily	☐ Unknown	☐ Other (specify):

B1.14.What is your religious denomination? (select one)

○ African Methodist
○ UCZ
○ 7th Day Adventist
○ Reformed Church
○ Baptist
○ New apostolic
○ CMML
○ Salvation Army
○ Jehovah's Witness (Watchtower)
○ Pentecostal
○ Presbyterian
○ Anglican
○ Catholic
○ Muslim
○ Hindu
○ Zionist
○ Baha'i
○ Other (Specify)_______________
○ None
○ Do not know
○ Refused

B1.15. What is your marital status? (select one)

○ Single, Never married
○ Married, single wife/husband
○ Married, multiple wives
○Widowed
○ Divorced
○ Separated
○ Refused

B1.16. What is your highest level of education? (select one)

○ No formal education
○ Primary school
○ Secondary school
○ College
○ Graduate degree
○ Professional degree
○ Refused

B1.17. What is your relationship to the head of household? (select one)

○ Head
○ Wife or Husband
○ Son or Daughter
○ Son-in-Law or Daughter-in-Law
○ Grandchild
○ Parent
○ Parent-in-Law
○ Brother or Sister
○ Niece/Nephew by blood
○ Niece/Nephew by marriage
○ Other Relative
○ Adopted/Foster/Stepchild
○ Not Related
○ Do not know
○ Refused

B1.18 Do you or any members of your household own any of the following? (Read list, record item if owned. Multiple responses possible)

☐ Bicycle
☐ Motorcycle/Motor scooter
☐ Car or truck
☐ Animal-drawn cart
☐ Boat with a motor
☐ Banana boat (without a motor)
☐ Watch
☐ Radio
☐ Television
☐ Refrigerator
☐ Agricultural land
☐ House
☐ Mobile phone
☐ Bed
☐ Refused

If province is Lusaka (B.0.3), continue with B1.19. If province is NOT Lusaka, skip to B1.28

Satisfaction: *We would now like to ask you a few questions about your experiences with the clinic and your HIV care providers. Please remember that this survey and your responses are private and confidential. Please indicate whether you agree or disagree with the following statements.*

In ODK, include the following graphic for "Agree" 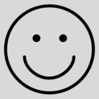 **, this graphic for "neither agree nor disagree"** 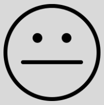 and this graphic for "disagree" 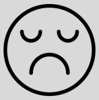

B1.19 I am satisfied that my providers at the [original clinic] have been taking care of me.	○ Agree<br>○ Neither Agree nor Disagree→go to B1.20<br>○ Disagree<br>○ Refused→go to B1.20
B1.19A. How strongly do you [agree/disagree]?	○ Somewhat [Agree/Disagree]<br>○ Strongly [Agree/Disagree]
B1.20 My providers [at X clinic] explain the reason(s) for any medical tests.	○ Agree<br>○ Neither Agree nor Disagree→go to B1.21<br>○ Disagree<br>○ Refused→go to B1.21
B1.20A. How strongly do you [agree/disagree]?	○ Somewhat [Agree/Disagree]<br>○ Strongly [Agree/Disagree]
B1.21 My providers [at X clinic] explain things in a way that is easy for me to understand	○ Agree<br>○ Neither Agree nor Disagree→go to B1.22<br>○ Disagree<br>○ Refused→go to B1.22

B1.21A. How strongly do you [agree/disagree]?	○ Somewhat [Agree/Disagree]<br>○ Strongly [Agree/Disagree]
B1.22 I am confident of my medical providers' knowledge and skills at the [at X clinic].	○ Agree<br>○ Neither Agree nor Disagree→go to B1.23<br>○ Disagree<br>○ Refused→go to B1.23
B1.22A. How strongly do you [agree/disagree]?	○ Somewhat [Agree/Disagree]<br>○ Strongly [Agree/Disagree]
B1.23 My medical providers [at X clinic] show respect for what I have to say.	○ Agree<br>○ Neither Agree nor Disagree→go to B1.24<br>○ Disagree<br>○ Refused→go to B1.24
B1.23A. How strongly do you [agree/disagree]?	○ Somewhat [Agree/Disagree]<br>○ Strongly [Agree/Disagree]
B1.24 My medical providers [at X clinic] listen carefully to me.	○ Agree<br>○ Neither Agree nor Disagree→go to B1.25<br>○ Disagree<br>○ Refused→go to B1.25
B1.24A. How strongly do you [agree/disagree]?	○ Somewhat [Agree/Disagree]<br>○ Strongly [Agree/Disagree]
B1.25 My medical providers [at X clinic] really care about me as a person.	○ Agree<br>○ Neither Agree nor Disagree→go to B1.26<br>○ Disagree<br>○ Refused→go to B1.26
B1.25A. How strongly do you [agree/disagree]?	○ Somewhat [Agree/Disagree]<br>○ Strongly [Agree/Disagree]
B1.26 My medical providers [at X clinic] encourage me to talk about all my health concerns	○ Agree<br>○ Neither Agree nor Disagree→go to B1.27<br>○ Disagree<br>○ Refused→go to B1.27
B1.26A. How strongly do you [agree/disagree]?	○ Somewhat [Agree/Disagree]<br>○ Strongly [Agree/Disagree]
B1.27 My medical providers [at X clinic] spend enough time with me.	○ Agree<br>○ Neither Agree nor Disagree→go to B1.28<br>○ Disagree<br>○ Refused→go to B1.28
B1.27A. How strongly do you [agree/disagree]?	○ Somewhat [Agree/Disagree]<br>○ Strongly [Agree/Disagree]
**Audit-C Scale**	
B1.28 How often do you have a drink containing alcohol?	○ Never<br>○ 1 time a month or less<br>○ 2-4 times a month<br>○ 2-3 times a week<br>○ 4 or more times a week<br>○ Refused
B1.29 How many standard drinks containing alcohol do you have on a typical day?	○ 0<br>○ 1 or 2<br>○ 3 or 4<br>○ 5 or 6<br>○ 7 to 9<br>○ 10 or more

B1.30 How often do you have six or more drinks on one occasion?	○ Never<br>○ Less than monthly<br>○ Monthly<br>○ Weekly<br>○ Daily or almost daily<br>○ Refused
**Domestic Violence**. Intro: *Now I'm going to give you a couple of scenarios and I want to know if you agree with the statements.*	
B1.31 If someone in the household misuses money it is acceptable to beat him/her	○ Yes<br>○ No<br>○ Refused
B1.32. In my household if a wife comes home late without the permission of the husband, she will be beaten	○ Yes<br>○ No<br>○ Refused
**Stigma:** *We would now like to ask you a few questions about your experiences living with HIV and opinions. Please remember that this survey and your responses are private and confidential. Please indicate whether you agree or disagree with the following statements.*	
B1.33 I think less of myself because of my HIV status	○ Agree<br>○ Neither Agree nor Disagree→go to B1.34<br>○ Disagree<br>○ Refused→go to B1.34
B1.33A. How strongly do you [agree/disagree]?	○ Somewhat [Agree/Disagree]<br>○ Strongly [Agree/Disagree]
B1.34 I have felt ashamed because of my HIV status	○ Agree<br>○ Neither Agree nor Disagree→go to B1.35<br>○ Disagree<br>○ Refused→go to B1.35
B1.34A. How strongly do you [agree/disagree]?	○ Somewhat [Agree/Disagree]<br>○ Strongly [Agree/Disagree]
B1.35 I have lost respect or standing in the community because of my HIV status	○ Agree<br>○ Neither Agree nor Disagree→go to B1.36<br>○ Disagree<br>○ Refused→go to B1.36
B1.35A. How strongly do you [agree/disagree]?	○ Somewhat [Agree/Disagree]<br>○ Strongly [Agree/Disagree]
B1.36 People hesitate to start HIV care because they are afraid others will learn their HIV status	○ Agree<br>○ Neither Agree nor Disagree→go to B1.37<br>○ Disagree<br>○ Refused→go to B1.37
B1.36A. How strongly do you [agree/disagree]?	○ Somewhat [Agree/Disagree]<br>○ Strongly [Agree/Disagree]
B1.37 People hesitate to start HIV care because they may be talked badly about	○ Agree<br>○ Neither Agree nor Disagree→go to B1.38<br>○ Disagree<br>○ Refused→go to B1.38
B1.37A. How strongly do you [agree/disagree]?	○ Somewhat [Agree/Disagree]<br>○ Strongly [Agree/Disagree]
B1.38 People hesitate to start HIV care because they may lose respect or standing	○ Agree<br>○ Neither Agree nor Disagree→go to B1.39<br>○ Disagree<br>○ Refused→go to B1.39
B1.38A. How strongly do you [agree/disagree]?	○ Somewhat [Agree/Disagree]<br>○ Strongly [Agree/Disagree]
B1.39 People hesitate to start HIV care because they may be verbally insulted, harassed, or threatened	○ Agree<br>○ Neither Agree nor Disagree→go to B1.40<br>○ Disagree<br>○ Refused→go to B1.40
B1.39A. How strongly do you [agree/disagree]?	○ Somewhat [Agree/Disagree]<br>○ Strongly [Agree/Disagree]
B1.40 I am, or have been, reluctant to access ARV drugs in the community where I live	○ Agree<br>○ Neither Agree nor Disagree→go to B1.41<br>○ Disagree<br>○ Refused→go to B1.41
B1.40A. How strongly do you [agree/disagree]?	○ Somewhat [Agree/Disagree]<br>○ Strongly [Agree/Disagree]
B1.41 People living with HIV who are taking ART are treated better by others than people living with HIV who are not taking ART	○ Agree<br>○ Neither Agree nor Disagree→go to B1.42<br>○ Disagree<br>○ Refused→go to B1.42
B1.41A. How strongly do you [agree/disagree]?	○ Somewhat [Agree/Disagree]<br>○ Strongly [Agree/Disagree]

Please tell us how often the following things have happened to you, or whether you think they have happened to you, because of (as a result of) your HIV status in the last 12 months?		
B1.42 People have talked badly about me because of my HIV status	☐	**Response categories:**<br>1 - Never<br>2 - Once<br>3 - A few times<br>4 - Often<br>5 - Not in the last 12 months, but have experienced before<br>6 - Not applicable because no-one knows my status<br>7- refused
B1.43 Healthcare workers talked badly about me because of my HIV status	☐	
B1.44 I have been verbally insulted, harassed or threatened because of my HIV status	☐	
B1.45 I have been physically assaulted because of my HIV status	☐	
B1.46 A health worker disclosed my HIV status without my permission	☐	
B1.47 I have lost respect or standing in the community because of my HIV status	☐	
B1.48 I have felt that people have not wanted to sit next to me for example on public transport, at church or in a waiting room because of my HIV status	☐	
B1.49 Someone disclosed my HIV status without my permission	☐	
B1.50 I confronted, challenged, or educated someone who was stigmatizing and/or discriminating against me	☐	
**If Lusaka Province selected in B.0.3, continue with B1.51. If Province is not Lusaka, GO TO SECTION B.4**		

B1.51. Was blood drawn for viral load testing?		
○Yes		
○No	B1.51A. If no, why not?	☐ Patient refused<br>☐ Attempted, unable to draw<br>☐ Other, specify:<br>☐ Not applicable: no indication patient has started ART
B1.52. Was PIMA CD4 testing carried out? (this question applies to patients for patients never on ART at the time of contact with patient)		
○Yes	B1.52A If yes, what was the PIMA CD4 result?	☐☐☐☐ cells/mm^3
○No	B1.52B. If no, why not?	☐ Patient refused<br>☐ Attempted, unable to obtain blood<br>☐ Blood attempted but PIMA assay failed<br>☐ Other, specify:
**GO TO SECTION B.4**		

SECTION B.2: TRACER COMMUNICATES WITH INFORMANT(S) WHO KNOWS THE PATIENT (NO COMMUNICATION WITH PATIENT)

B.2.1 As of this interview, has field tracing ever occurred for this patient?	○Yes<br>○No

B.2.2. Interview type: *[pick one]*	☐ in-person<br>☐ phone	B.2.3. Informant Interview date.	☐☐ - ☐☐☐ - ☐☐<br>DAY(*dd*) MONTH (*mm*) YEAR(*yy*)

B.2.4. Confirm identity of the patient using as many identifiers as possible. *Mark all that apply.*

☐ Name ☐ Age ☐ Gender ☐ Height ☐ Location or residence ☐ Occupation ☐ Clinic number or medical papers
☐ Marital Status ☐ Common Name

B.2.5. What is your relationship to [state patient's name]? *[Pick one]*

☐ Spouse	☐ Grandparent	☐ Parent	☐ Local leader	☐ Other relative
☐ Neighbor	☐ Child	☐ Friend	☐ Brother or sister	☐ Other, specify:

B.2.6. As far as you are aware, is the patient alive?

○Yes	B.2.6.A. When was the last time you or someone else you know had contact with the patient?	☐☐ - ☐☐☐ - ☐☐<br>DAY(*dd*) MONTH (*mm*) YEAR(*yy*)
	B.2.6.B. Did he/she move usual residence?	○ Yes ○ No ○ Unknown
	**GO TO SECTION B.4**	
○ No, patient has died	B.2.6.C. When did the patient pass away?	☐☐ - ☐☐☐ - ☐☐<br>DAY(*dd*) MONTH (*mmm*) YEAR(*yy*)
	B.2.6.D. How did he/she die? *Mark one.*	○ Suicide → go to **Section B.4**<br>○ Disease or illness<br>○ Injury, accident, or trauma → go to **Section B.4**<br>○ Relating to childbirth→ go to **Section B.4**<br>○ Don't know/declines to answer→ go to **Section B.4**<br>○ Other, specify:______________ → go to **Section B.4**
	**If answer to B.2.6.D is "disease or illness":**	
	B.2.6.E. Did the patient see a doctor, nurse or other non-traditional health professional in the two weeks before he or she died?	○ Yes<br>○ No→go to **Section B.4**<br>○ Unknown → go to **Section B.4**<br>○ Refused → go to **Section B.4**<br>○ Not Asked→ go to **Section B.4**
	B.2.6.F. What did the health care providers say the patient was sick with?	○ Known, Specify:________________________<br>○ Unknown<br>○ Refused<br>○ Not Asked

GO TO SECTION B.4

SECTION B.3: TRACER UNABLE TO COMMUNICATE WITH PATIENT OR INFORMANT

B.3.1. Is there a record of patient death in any local death registry?	○ Yes<br>○ No evidence of death in registry<br>○ No registry available	B.3.2. If there is a record of a death, when did the patient die?	☐☐ - ☐☐☐ - ☐☐<br>DAY(*dd*) MONTH (*mmm*) YEAR(*yy*)

B.3.3. How far was the tracer able to get in the physical tracing of the patient? (For the questions below, "site" refers to smallest geographic unit available to tracer in clinic records. "Residence" is the actual building or dwelling where the patient was reported to have stayed)

○ Site found and residence found	B.3.3.A. Why was no further information about the patient obtained?	○ No one available to speak to<br>○ None of the available persons were willing to talk<br>○ None of the available persons knew the patient<br>○ No one willing to talk about the patient even though they know him/her<br>○ No one knows the patient's current vital status or care status even though they know him/her<br>○ Other, specify: _______________
○ Site found but residence not found	B.3.3.B. Why were you unable to find the residence?	○ Site refers to an area that is too big to be able to search for patient<br>○ No one available to speak to<br>○ None of the available persons was willing to talk<br>○ None of the available persons knew the patient<br>○ No one willing to talk about residence even though they know him/her<br>○ No one knows about residence even though they know him/her<br>○ Other, specify: _______________
○ Site not found and therefore residence of patient was not found	B.3.3.C. Why were you unable to find the site given by the clinic?	○ Site does not exist<br>○ Site inaccessible (e.g.,flood)<br>○ Site data not legible enough to understand<br>○ Other, specify:________________
○ Phone tracing only	B.3.3.D. Why was phone tracing unsuccessful?	○ The phone number is invalid<br>○ No one answered the phone<br>○ The person answering the phone does not know the patient<br>○ The person answering the phone refused to provide information about the patient<br>○ The patients' file has no contact/phone numbers or details<br>○ Other, specify: _____________

Go To Section B.4

Section B.4: TRACER FEEDBACK

B.4.1. What difficulties or challenges did you face in tracing this patient?			
B.4.2. What suggestions or ideas do you have to improve this process or protocol?			
B.4.3a. How many persons were questioned by phone while looking for this patient?	☐☐		
B.4.3b. How many persons were questioned in person while looking for this patient?	☐☐		
B.4.4. How many hours were spent by phone looking for this patient?	☐☐:☐☐<br>Hours *(hh)* : Minutes *(mm)*	B.4.5. How many hours were spent in person looking for this patient?	☐☐:☐☐<br>Hours *(hh)* : Minutes *(mm)*
B.4.6. How many trips were made looking for this patient?	☐☐	○ Not applicable, phone tracking only	
B.4.7. Does patient have a new site or residence?	○ Yes: Address known → *Update contact form*<br>○ Yes: He/she has moved but do not know where<br>○ No<br>○ Don't Know<br>○ Not applicable (died)	B.4.8. Does the patient have a new phone contact number?	○ Yes: *Update contact form*<br>○ No<br>○ Don't Know<br>○ Not applicable (died)
B.4.9. What ART treatment facility is closest to the patient's primary place of residence?			
B.4.10. What types of transport did you use in your efforts to trace this patient? *[mark all that apply]*	○ Walking on foot<br>○ Bicycle<br>○ Motorbike<br>○ Public Transport<br>○ Study Vehicle<br>○ Private hired transport<br>○ Ox Cart<br>○ None (it was phone only tracing)<br>○ Other: _________________ (specify)		
