# Supplementary material for: Patient-reported Reasons for Stopping Care or Switching Clinics in Zambia: A Multisite, Regionally Representative Estimate Using a Multistage Sampling-based Approach in Zambia
Source: Clin Infect Dis. 2020 Oct 3;73(7):e2294–302. doi: 10.1093/cid/ciaa1501 (PMC8492131; doi:10.1093/cid/ciaa1501)
Supplement: ciaa1501_suppl_Supplementary_Appendix_S2 [file ciaa1501_suppl_supplementary_appendix_s2.pdf]

Resources required to ascertain the outcomes of HIV-positive patients lost to follow-up (LTFU) – BetterInfo Toolkit

Background:

The Better Information for Health in Zambia (BetterInfo) Study used the sampling-based approach to understand the true outcomes (retention and mortality) of HIV Lost to Follow-up patents. In order to ascertain the true outcomes, the BetterInfo Study used paper, phone and field tracking. *Paper tracking* is considered the first step and '*basic option'* for tracking LTFU patients in resource-limited settings because it is cost-effective. This option entailed establishing a sample of HIV "lost" patients to be tracked, reviewing their files/charts in the health facilities and completing a simple questionnaire to capture vital and HIV in-care status. *Phone Tracking* is the second step which involves contacting the patient or patients' next-of-kin using telephonic communication. The aim of phone tracking was to obtain as much information as possible including obtaining any updated patient and/or treatment supporter/buddy contact information (phone numbers, geographic information); determine the patient's outcomes (alive or dead, in-care or out-of-care and reasons for out-of-care or transfer); and to document what had been learnt through the tracking process. *Field Tracking* is the third and final step which involved the tracker going into the community (by walking, bicycle, motor bike, and/or public transport) to try to locate the "lost" patient in person. The aim was to obtain any updated patient and/or treatment supporter/buddy contact information (phone numbers, geographic information); determine the patient's outcomes (alive or dead, in-care or out-of-care and reasons for out-of-care or transfer); administer questionnaires; and if applicable, obtain biological specimens.

Additionally, the BetterInfo Study also sought to document the implementation process of the sampling-based approach so as to ascertain the time and cost resources required to support an efficient replication of the sampling-based approach in similar settings.

Methods:

The measurement of *'time spent tracking the LTFU'* was embedded in the data collection forms which were completed by the trackers. We also monitored all tracking activities using an Enterprise Resource Planning (ERP) online financial management system to build cost estimates for future implementers. More specifically, efficient tracking of patients lost from HIV care required resources such as, staff, computer tablets, mobile phones and mobile talk time, transportation (public transport, motorbikes, bicycles), tracker supplies (back packs, rain suits/rain coats and umbrellas), and testing supplies (reagents, syringes, gloves, cool boxes etc.) and HIV Viral Load and Resistance test kits.

Results:

BetterInfo Study was able to ascertain about 50% of the patient outcomes after 7 days of paper tracing attempt. See table 1:

Table 1: Outcomes ascertained since the first tracking attempt

Days since first tracking attempt	Patient Outcome ascertained N (cumulative %)
0	1337 (31)
7	2089 (48)
14	2461 (57)
21	2637 (61)
60	3011 (69)
90	3124 (72)
180	3226 (74)
>180	3247 (75)

Generally, there was variation in time taken to track lost patients by paper tracking only, paper and phone tracking only and paper, phone and field tracking. The average time taken to paper track lost patients was 54 minutes, 60.5 minutes for paper and phone tracking only and 300.5 minutes for paper, phone and field tracking. See Table 2:

Table 2: Average time for Paper, Phone and Field Tracking of LTFU

Tracking Level	Average Time for Tracking
Paper Tracking Only	54 minutes
Paper and Phone Tracking Only	60.5 minutes (1 hour 05 minutes).
Paper, Phone and Field Tracking	300.5 minutes (5 hour 05 minutes).

Similarly, there was variation in the minimum costs required to ascertain the true outcomes of HIV lost patients. The patient cost per outcome ascertained during paper tracking only was $ 916 (and comprised of training, human resources and computer tablet resources); paper and phone tracking only was $ 954 (and comprised of training, human resources, computer tablet, mobile phones and talk time resources) and paper, phone and field tracking was $ 5,469 (and comprised of training, human resources, computer tablet, mobile phones, talk time, tracker supplies, per diem, lunch allowances, transportation and costs of viral load and HIV drug resistance). See Table 3:

Table 3: Cost per Outcome ascertained during Paper, Phone and Field Tracking of LTFU

Tracing Level	Cost per Outcome ascertained ($)
Paper Tracking Only	$ 916
Paper and Phone Tracking Only	$ 954
Paper, Phone and Field Tracking	$ 5,469

Reference

Centre for Infectious Disease Research in Zambia (2017): A Toolkit for a Sampling-Based Approach to Understand HIV Treatment Outcomes – Better Information for Health in Zambia (BetterInfo) Study, November 2017, Lusaka, Zambia. http://www.cidrz.org/wp-content/toolkits/betterinfo/
